# Validation of Correction Algorithms for Near-IR Analysis of Human Milk in an Independent Sample Set—Effect of Pasteurization

**DOI:** 10.3390/nu8030119

**Published:** 2016-02-26

**Authors:** Gynter Kotrri, Gerhard Fusch, Celia Kwan, Dasol Choi, Arum Choi, Nisreen Al Kafi, Niels Rochow, Christoph Fusch

**Affiliations:** Division of Neonatology, Department of Pediatrics, McMaster University, 1280 Main Street West, Room HSC-4F5, Hamilton, ON L8S 4K1, Canada; gynter1994@hotmail.com (G.K.); gefusch@mcmaster.ca (G.F.); kwanc4@mcmaster.ca (C.K.); choi7453@naver.com (D.C.); choiar@mcmaster.ca (A.C.); nkafi@yahoo.com (N.K.); nielsrochow@gmail.com (N.R.)

**Keywords:** milk, analyzer, fat, protein, lactose, term, preterm, nutrition, donor milk

## Abstract

Commercial infrared (IR) milk analyzers are being increasingly used in research settings for the macronutrient measurement of breast milk (BM) prior to its target fortification. These devices, however, may not provide reliable measurement if not properly calibrated. In the current study, we tested a correction algorithm for a Near-IR milk analyzer (Unity SpectraStar, Brookfield, CT, USA) for fat and protein measurements, and examined the effect of pasteurization on the IR matrix and the stability of fat, protein, and lactose. Measurement values generated through Near-IR analysis were compared against those obtained through chemical reference methods to test the correction algorithm for the Near-IR milk analyzer. Macronutrient levels were compared between unpasteurized and pasteurized milk samples to determine the effect of pasteurization on macronutrient stability. The correction algorithm generated for our device was found to be valid for unpasteurized and pasteurized BM. Pasteurization had no effect on the macronutrient levels and the IR matrix of BM. These results show that fat and protein content can be accurately measured and monitored for unpasteurized and pasteurized BM. Of additional importance is the implication that donated human milk, generally low in protein content, has the potential to be target fortified.

## 1. Introduction

Breast milk is the nutritional standard for preterm infants. Its unique composition of essential nutrients is crucial for the development of the neonate’s premature host defense system, as well as the improvement of long-term neurodevelopmental outcome [1]. Due to the fact that the macronutrient content is inherently too low for the nutritional needs of preterm infants, breast milk is commonly fortified with a standard milk fortifier. However, such an approach does not take into consideration the natural variation of macronutrient content in human milk and may fail to compensate for cases where native milk has below average macronutrient levels [2,3]. The subsequent feeding of neonates with milk containing suboptimal macronutrient levels can lead to postnatal growth restriction [4].

Recently, the target fortification of breast milk on the basis of measured macronutrient content has shown the potential to reduce macronutrient deficits and achieve predicted growth in preterm infants [5]. The clinical implementation of this procedure requires the optimal, simple and rapid measurement of macronutrient content in breast milk. Commercially available infrared (IR) milk analyzers originally developed for use in the dairy industry have been introduced for this purpose. However, due to the significant differences in matrix composition between human and cow milk, IR measurement read-outs of breast milk may not be accurate without previous validation [6]. In a recent study, this shortcoming was addressed for the measurement of fat and protein levels with a Near-IR milk analyzer. A correction algorithm was created by correlating a large number of Near-IR measurement results against those generated through validated chemical reference methods [6]. This approach has never been tested in an independent set of breast milk samples.

Another requisite necessary for target fortification of breast milk is the precise knowledge of the stability of its macronutrient content. This is because, in the clinical setting, the preparation of breast milk for feeding does not happen immediately following its collection. During this time span between its collection and feeding, breast milk may be subjected to pasteurization to ensure its microbiological quality. This is also the case with donated human milk fed to infants when the mother’s own milk is insufficient or unavailable [7]. Inconsistencies exist in the current literature regarding the effect of pasteurization on macronutrient levels in breast milk [8,9,10,11,12]. Furthermore, pasteurization could alter the matrix of human milk and hence affect the accuracy and precision of the readout of IR analyzers.

The aim of the current study was to validate the newly proposed correction algorithm for Near-IR milk analyzers for fat and protein content measurement in an independent set of native human milk samples. Another aim of the study was to test the ability of the correction algorithm to generate reliable measurement for breast milk subjected to Holder pasteurization. Additionally, the stability of fat, protein, and lactose content and of their respective matrices were examined as breast milk was subjected to Holder pasteurization.

## 2. Experimental Section

### 2.1. Study Design and Sample Collection

This was a prospective study conducted at McMaster Children’s Hospital’s level 3 Neonatal Intensive Care Unit (NICU) in Hamilton, Ontario, Canada. The study consisted of three components: (1) validation of a previously-published correction algorithm for Near-IR (Unity) milk analyzers in an independent set of unpasteurized breast milk samples; (2) assessment of the reliability of the correction algorithm in an independent sample set of breast milk samples subjected to Holder pasteurization; and (3) determination of the impact of Holder pasteurization on macronutrient content stability in human milk as measured by IR and chemical reference methods. Human milk samples used in this study were generated through the pooling of residual frozen breast milk from various mothers. The study was approved by the Hamilton Integrated Research Ethics Board as a quality assurance study. Mothers donated leftover breast milk samples after discharge of their child explicitly for research purposes to the McMaster Neonatal Research Lab. For all samples, oral consent was given to the lactation consultants.

### 2.2. Measurement Methods for Fat, Protein, and Lactose in Breast Milk

Two methods of measurement were used throughout the study to determine macronutrient content levels in breast milk: chemical and Near-IR analysis. Chemical analyses of milk samples were performed using validated micro-methods, which required less than 1.5 mL of sample volume for all three macronutrients. These methods consisted of a modified ether Mojonnier extraction (1.0 mL) for fat, elemental analysis (0.260 mL) for protein, and ultra-performance liquid-chromatography tandem mass spectrometry (UPLC MS/MS) (0.100 mL) for lactose with corresponding coefficients of variation of 1.7%, 1.8%, and 2.3%, respectively [13,14]. Near-IR analysis was done through the SpectraStar Near-IR milk analyzer (Model 2400 RTW, Unity Scientific, Brookfield, CT, USA). In order to provide reliable measurement of fat and protein concentration in breast milk, a correction algorithm was generated by plotting Near-IR measurement values against those generated through chemical reference methods. The inverse linear function of each of the resulting regression equations was used as the correction algorithm for readout generated by the Near-IR milk analyzer [6]. In the present study, all measurement values produced by the Near-IR milk analyzer have been adjusted using this correction algorithm [6].

### 2.3. Preparation of the Sample Set

**Figure 1:**
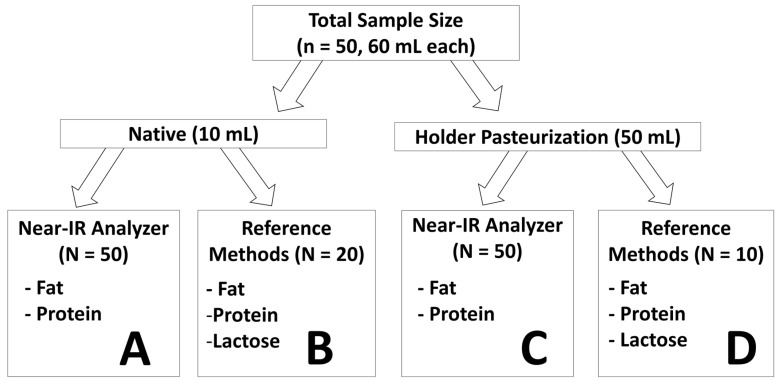
Study Design: To validate the Near-IR human milk analyzer in unpasteurized breast milk, group B samples were compared to the corresponding group A samples. The correction algorithm was also tested for pasteurized breast milk, through a comparison of all group D samples with the corresponding samples in group C. The effect of pasteurization on macronutrient levels was determined through two separate comparisons: one between groups A and C, and another between all group D samples and the corresponding samples in group B.

Fifty distinct samples (V = 60 mL) with different macronutrient contents were generated through thawing and subsequent pooling of residual, frozen breast milk from various mothers (Figure 1). Each sample was aliquoted into a 10 mL and 50 mL sample. The 50 mL samples were subjected to Holder pasteurization taking place in two separate locations: Toronto Milk Bank at Mount Sinai Hospital (*n* = 10) and Neonatal Intensive Care Unit at McMaster Children’s Hospital (*n* = 40). Samples were subjected to heating for approximately 30 min until a temperature of 62.5 °C was reached (Figure S1).

This temperature was maintained for a period of 30 min and was subsequently followed by a cool-down phase where the milk sample was placed in an ice bath until reaching a temperature of 4 °C (Figure S1). Pasteurized and unpasteurized samples were then aliquoted (V = 1.5 mL each) and subsequently stored at −20 °C temperature. For analysis, aliquoted samples were thawed at 37 °C for 5 min and homogenized using an ultrasonic vibrator (VCX 130; Chemical Instruments AB; Sollentuna, Sweden) for 15 s. All samples, pasteurized and unpasteurized, were measured using Near-IR analysis for concentrations of fat and protein (Groups A and C, Figure 1). From the 50 unpasteurized 10 mL-samples, a subset of 20 samples (Group B) was selected to measure fat, protein, and lactose content through chemical analysis. Furthermore, from this subset of 20 unpasteurized samples ten corresponding, but pasteurized counterparts, each originating from the same 60 mL sample (Group D), were also measured with chemical analysis to determine fat, protein, and lactose concentration. All analyses (Near-IR and chemical reference methods) of breast milk were conducted as a single measurement.

### 2.4. Component 1: Validation of the Near-IR Milk Analyzer

The validation of the correction algorithm for Near-IR macronutrient content measurement was done for unpasteurized milk samples. The fat and protein measurement values generated by the Near-IR milk analyzer were transformed using the correction algorithm. These corrected values in unpasteurized samples (subset of Group A) were compared against the values obtained through chemical reference methods (Group B, *n* = 20).

#### 2.4.1. Component 2: Assessment of the Reliability in Pasteurized Breast Milk

The correction algorithm was tested for pasteurized breast milk through the comparison of corrected Near-IR (subset of Group C) and chemical reference method measurement values (Group D) for the 10 pasteurized samples measured through both methods.

#### 2.4.2. Component 3: Effect of Pasteurization

The effect of pasteurization on macronutrient levels in breast milk was examined through the comparison of corresponding samples measured with identical methods. Each pasteurized and unpasteurized sample being compared originated from the same original, pooled 60 mL sample of breast milk. In 50 samples, 10 mL of unpasteurized milk measured with Near-IR (Group A) was compared against 50 mL of pasteurized milk (Group C). Similarly, 10 samples of pasteurized milk measured with chemical reference methods (Group D) were also compared against the corresponding unpasteurized sample measured with identical methods (subset of Group B).

### 2.5. Statistical Analysis

To compare the results of macronutrient content measurement of unpasteurized and pasteurized milk with the Near-IR milk analyzer against chemical methods, correlation analysis was performed that included the calculation of Pearson correlation coefficient using Microsoft Excel 2010 (Redmond, WA, USA). Additionally, linear regression analysis was computed. For the Bland–Altman plot, the differences between two methods were graphed against the reference method instead of using the mean value of both methods. This approach is methodologically appropriate when comparing against a true reference or standard [15]. The mean precision for fat and protein determination was calculated by taking the standard deviation of the differences between Near-IR and chemical reference method values.

## 3. Results

**Figure 2:**
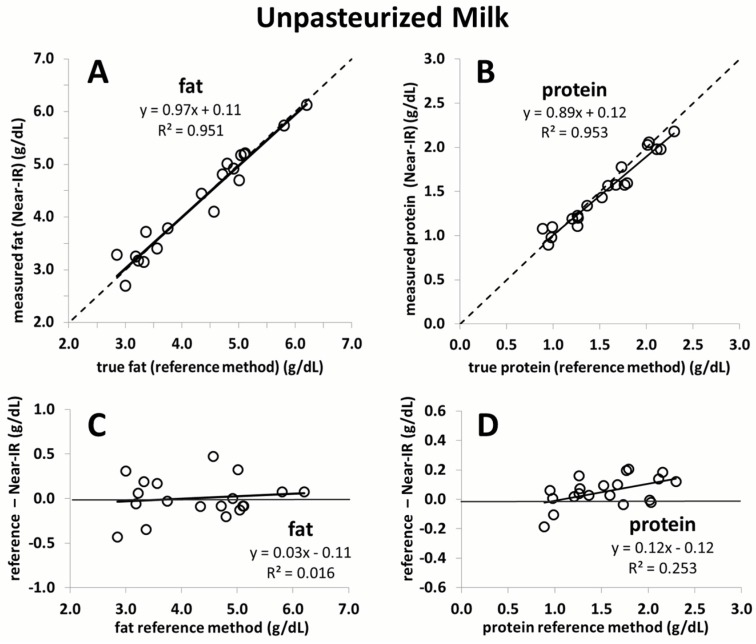
Fat (**A**) (*n* = 20) and protein (**B**) (*n* = 20) values as measured through chemical reference methods correlated against corrected Near-IR human milk analyzer values. Bland–Altman plots for fat (**C**) and protein (**D**) indicating the difference obtained by the reference method (*x*-axis) and the corrected Near-IR human milk analyzer values represented in *y*-axis.

**Figure 3:**
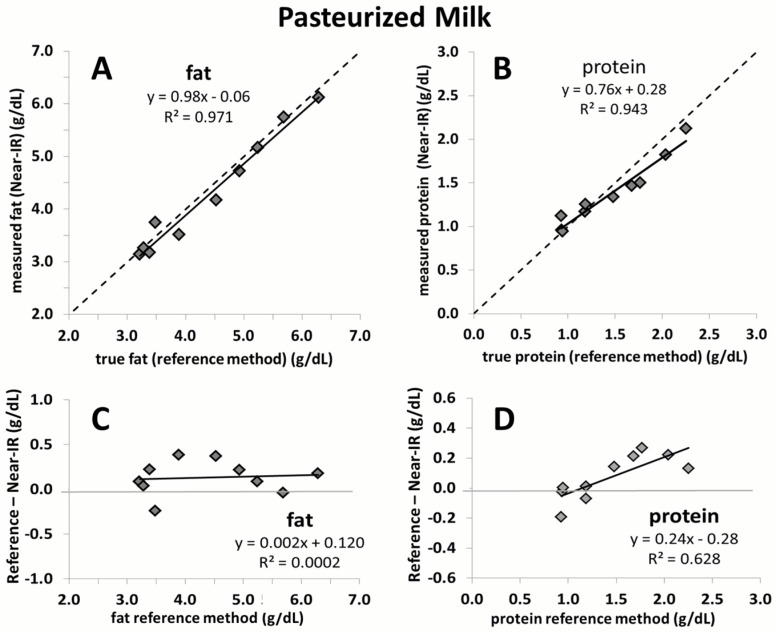
Fat (**A**) (*n* = 10) and protein (**B**) (*n* = 10) values in pasteurized breast milk as measured through chemical reference methods correlated against corrected Near-IR human milk analyzer values; Bland–Altman plots for fat (**C**) and protein (**D**) indicating the difference in pasteurized breast milk obtained by the reference method (*x*-axis) and the corrected Near-IR human milk analyzer values represented in *y*-axis.

**Figure 4:**
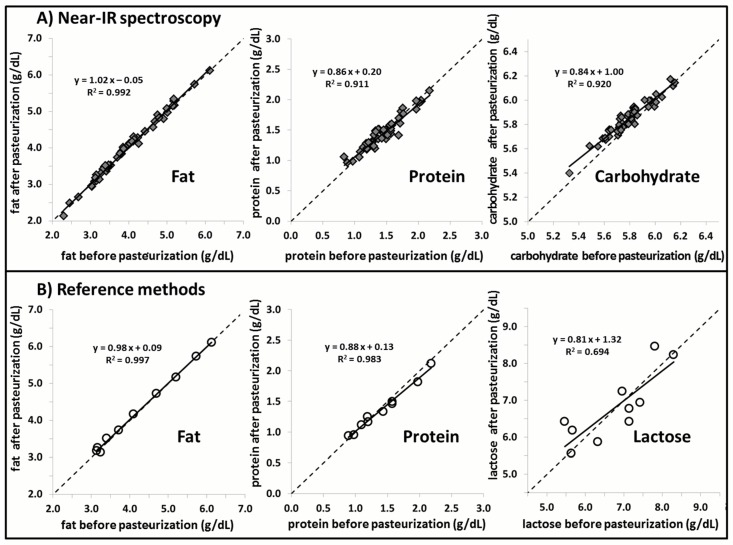
(**A**) shows the correlation between samples of pasteurized and unpasteurized breast milk (*n* = 50) as measured through Near-IR milk analyzers and subsequently adjusted with the correction algorithm; (**B**) shows a similar correlation between pasteurized and unpasteurized samples (*n* = 10) as measured through validated chemical reference methods.

Figure 2 and Figure 3 show a strong correlation between measurements generated by the Near-IR human milk analyzer (following application of the correction algorithm) against those generated through reference methods for fat and protein content (*n* = 20). The mean precision is ±0.2 g/dL for fat determination and ±0.1 g/dL for protein determination. The correlation between fat and protein shown includes both unpasteurized (*n* = 20), and pasteurized (*n* = 10) breast milk. Figure 4 shows high agreement between macronutrient content of pasteurized and unpasteurized samples.

## 4. Discussion

The current study confirmed that a recently proposed correction algorithm for Near-IR analyzers is capable of producing reliable and accurate measurements of fat and protein content in human milk. This was shown in an independent sample set consisting of unpasteurized and pasteurized human milk. Moreover, the study results demonstrate that the macronutrient content, as well as the chemical matrix, remains stable in breast milk following Holder pasteurization. The results of this study affirm the feasibility of rapid macronutrient measurement in pasteurized and unpasteurized breast milk and the subsequent monitoring of its macronutrient content.

This is the first study to validate a calibration algorithm for an IR milk analyzer in an independent sample set. Commercial IR milk analyzers, originally intended for measurement of dairy milk, may not be able to consistently produce accurate and reliable measurements of human milk. This is due to matrix differences that exist between dairy and human milk, such as significant levels of oligosaccharides in human milk, different fatty acid profiles, and changes in the ratio of casein-to-lactalbumin concentration. In a previous study, we conducted the calibration of Near-IR milk analyzers for the measurement of human milk [6]. Methodologically, this is the most reliable calibration of an IR milk analyzer for human milk to date. This is largely due to the fact that three obligatory components to generate a correction algorithm of milk analyzers for clinical purposes were carefully considered: (1) the use of chemical reference methods; (2) a sufficiently large sample set; and (3) human milk representative of the clinical setting. With respect to the first criteria, there is a lack of agreed-upon chemical reference methods for measurement of macronutrient content in human milk. We therefore previously made a strategic decision to develop and validate methods that are appropriate to measure macronutrient concentration in small volumes (<1.5 mL) of breast milk. These methods and their validation against standards of Association of Official Analytical Chemists (AOAC) have been described in detail in previous publications [13,14]. We feel confident that our established chemical reference methods can therefore be considered as a “golden” standard against which to compare the accuracy of the corrected IR measurement values [6]. While the spectroscopic measurement of the devices is quite precise, an error will be introduced if a correction algorithm is charged with an imprecision. We believe that the use of human milk analyzers for routine clinical decision-making should require that the devices have a random error (imprecision) of less than 2%. We ensured this through the use of a sufficiently large number of breast milk samples (*n* = 900). Moreover, these samples reflected the full range of gestational age and lactation periods seen in the clinical setting. The robustness of our correction algorithm is shown by its ability to generate reliable measurement in an independent set of breast milk samples in the current study. It is of interest to note that the smaller sample size in the current study led to a slightly narrower range of macronutrient concentration being covered when compared to the sample set used for calibration. However, the range of the current study still comprises most of the macronutrient concentration values observed in the calibration study (77% for fat, 72% for lactose, and 96% for protein) (Figure S2). Moreover, the samples in the present study used for validation are equidistant, whereas those used in the previous study to calibrate the device are not as evenly distributed and mostly concentrated in the centre of the regression line. The strength of the correction algorithm is further affirmed by its ability to generate accurate measurement results in pasteurized milk, for which it was not originally intended. While the validation of fat yielded nearly perfect correlation close to the line of identity, a slight deviation is seen for protein. This is likely due to the smaller sample size used (*n* = 10). Random error attributed to either the Near-IR or the chemical reference method will have a much greater impact on the slope of the line compared to a large sample size. Nevertheless, the error over the range of protein measurement in this case does not exceed 0.1 g/dL, which we believe is not clinically relevant.

It should be noted that the parameters of the calibration algorithm generated in our previous study and validated in this study might not be applicable for other Near-IR milk analyzers. Ideally, an independent calibration algorithm should be generated for each individual IR milk analyzer in use. There are various avenues that can be pursued to correct an offset present in milk analyzers. The first option, a primary recalibration, incorporates the measurement of a novel data set of IR and established chemical reference values. This process would need to be performed by the manufacturer. If this is not a possibility, the imprecision can be corrected through the application of the inverse function of the linear regression (as seen in our own evaluation) [6]. This option requires that the correction algorithm be implemented directly in the software of the device (*i.e.*, internal correction). If the software of the device does not allow for the adaptation of the correction algorithm, the read-out values generated by the milk analyzers can be adjusted with the correction algorithm using a table calculation program (*i.e.*, external correction). This alternative of device calibration can be implemented by all study groups through good clinical and laboratory practice.

To date, seven studies have investigated the validity of IR milk analyzers for macronutrient content measurement [16,17,18,19,20,21,22]. Out of these studies, only three have published independent calibrations of human milk analyzers [16,17,18]. In addition to their relatively small sample size, however, concerns must be raised with respect to chemical reference methods. This is especially true for the measurement of lactose where high pressure liquid chromatography (HPLC) is used. Though HPLC is the method of choice to detect lactose in cow milk, there is no agreed on HPLC setup to detect lactose in human milk. In fact, the gold standard guidelines of dairy industry clearly state for a certified HPLC setup (like ISO 22662:2007; IDF 198:2007) that the method is not applicable for cow’s milk to which oligosaccharides have been added [23]. Different from cow milk, however, human milk contains a large amount of oligosaccharides, which, inevitably, all contain a terminal lactose residue. The adequate use of HPLC for detection of lactose in breast milk will require a proper set-up and validation to account for the difference in the chemical matrix between human and dairy milk. This step has not been documented in previously published papers. For our research projects, we have expanded our method of lactose detection beyond the use of HPLC: we developed a method using tandem mass spectrometry (UPLC-MS/MS), which is specific for lactose measurement in human milk and shows no interference with oligosaccharides [14]. In a large sample set, we were able to show that lactose measured by both Near-IR and Mid-IR milk analyzers is poorly correlated with lactose content measured by LC-MS/MS [6]. Moreover, lactose levels measured in parallel by the two IR spectroscopic methods did not correlate at all, indicating that IR analyzers have methodological difficulties measuring lactose concentration. Interestingly, parallel measurements of fat and protein show a reasonably good agreement between both IR methods [6]. We believe that the large number of oligosaccharides is responsible to confound the lactose measurement. Therefore, we did not include the IR-measurement of lactose concentrations in the current study.

Stability of macronutrient (lactose, fat, and protein) content in human milk subjected to Holder pasteurization was also observed in the present study. These findings are consistent with previously published literature about the biochemical properties of the macronutrients of interest following Holder pasteurization. Espinosa-Martos *et al.* demonstrate through gas chromatography that the concentration of lactose in colostrum remains constant after pasteurization [24]. In the present study, we are able to verify a constant concentration of lactose in human milk following Holder pasteurization through UPLC-MS/MS. For fat, Holder pasteurization has been noted to produce elevated levels of free fatty acids in human milk [9]. The total amount of fat, however, when measured through gravimetric methods, has consistently been found to remain constant after pasteurization [9,10,11]. This was also demonstrated in the current study through the use of Mojonnier ether extraction, a method that ensures the minimal loss of fat while it is being prepared for weighing. This means that, although Holder pasteurization might induce the hydrolysis of triglycerides, the nutritional composition of fat in breast milk remains constant [10,11]. For protein, a similar alteration takes place when breast milk is pasteurized. Through elemental analysis, we demonstrated in the current study that the total amount of nitrogen in breast milk remains constant following pasteurization. In a similar finding, Valentine *et al.* did not note a significant difference between amino acid levels in breast milk before and after pasteurization [25]. From this we can deduce that the amount and distribution of amino acids remains constant following pasteurization. Although the structural integrity and the bioactivity of functional proteins may be compromised by prolonged elevated temperatures from Holder pasteurization, the nutritional composition of the protein content remains unchanged. This phenomenon is reaffirmed in the current study through Near-IR analysis, where the protein content before and after pasteurization remains constant. It should be noted that, although there is good agreement of lactose content before and after pasteurization of breast milk, the range differs between measurements conducted with Near-IR (5.0–6.5 g/dL) and UPLC-MS/MS (5.0–9.0 g/dL). This discrepancy in range of measurement output is likely to the inability of IR human milk analyzers to correctly measure lactose in human milk.

The stability of fat content, as measured through Near-IR milk analysis, is also demonstrated in the current study for breast milk following its pasteurization. IR spectroscopy is able to measure the concentration of a certain macronutrient based on the specific frequencies of light absorbed by these molecules in breast milk. These macronutrients, however, share many of the same functional groups that are targeted by Near-IR analysis, making it difficult to determine the true concentration of the macronutrient of interest. This effect of the chemical matrix on the Near-IR analysis could become more pronounced following pasteurization of breast milk. The current study was able to demonstrate that Holder pasteurization did not affect the measurement values of the Near-IR milk analyzer. The constancy of measurement with Near-IR analysis suggests that the changes in fatty acid profiles and protein bioactivity caused by Holder pasteurization have no effect on the chemical matrix of breast milk.

A similar study assessing the stability of the chemical matrix of breast milk was conducted by Vieira *et al.* This study used an infrared analyzer and notes significant reductions of fat and protein following pasteurization [8]. Unlike our study, where homogenization is conducted ultrasonically for 15 s, the authors do not mention homogenizing their milk samples prior to milk analysis. Homogenization before measurement reduces the adherence of fat to container walls, minimizes the fat globule diameter limit the impact of light scattering, and ensures fat is sufficiently distributed for a representative batch of milk to be drawn for analysis [6]. These factors may account for the differences to the fat concentration after pasteurization between both studies.

The current study has significant clinical implications when it comes to the use of donor milk for the feeding of preterm infants. Donor milk is currently recommended for preterm infants whose mothers are unable to produce sufficient amounts of breast milk. However, babies receiving donor breast milk have an increased risk of postnatal growth restriction [26]. This is due to the fact that donor milk is usually obtained from mothers who are in their late stages of lactation, which is generally low in protein [27]. The current study demonstrates that human milk analyzers, once properly adjusted through the generation of a correction algorithm, are capable of accurate and reliable measurement of fat and protein in pasteurized human milk. This is of relevance to donor breast milk, as it is routinely pasteurized following collection to ensure its microbiological safety. Moreover, the fat and protein content of breast milk was shown to remain constant after pasteurization, making feasible the continual monitoring of these macronutrients in donor milk. Consequently, this means that donor milk has the potential to be improved through target fortification in cases where mother’s own milk is unavailable.

## 5. Conclusions

The present study validated a correction algorithm for an IR milk analyzer in an independent sample of unpasteurized breast milk. Moreover, the algorithm was shown to be accurate for pasteurized human milk. We believe that recommendations provided for the validation process in this study provide a solid foundation for the generation of reliable correction algorithms of IR milk analyzers. Moreover, accurate IR measurement of pasteurized breast milk enables the use of donor milk for target fortification when mother’s own milk is unavailable.

## Supplementary Materials

The following are available online at http://www.mdpi.com/2072-6643/8/3/119/s1, Figure S1: Temperature profile of Holder pasteurization employed in the present study; Figure S2: Macronutrient distribution of samples used in calibration study.
