# Supplementary material for: Validation of Correction Algorithms for Near-IR Analysis of Human Milk in an Independent Sample Set—Effect of Pasteurization
Source: Nutrients. 2016 Feb 26;8(3):119. doi: 10.3390/nu8030119 (PMC4808849; doi:10.3390/nu8030119)
Supplement: Supplementary file 1 [file nutrients-08-00119-s001.docx]

Validation of Correction Algorithms for Near-IR Analysis of Human Milk in an Independent Sample Set—Effect of Pasteurization

Gynter Kotrri, Gerhard Fusch, Celia Kwan, Dasol Choi, Arum Choi, Nisreen Al Kafi,
Niels Rochow and Christoph Fusch


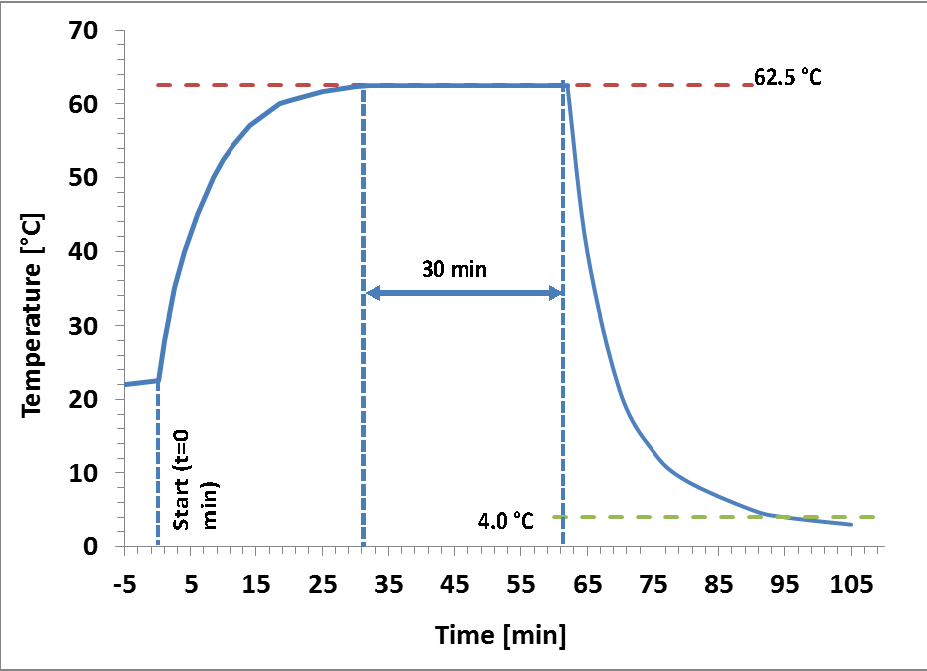


**Figure S1.** Temperature profile of Holder pasteurization of 50 mL breast milk samples.


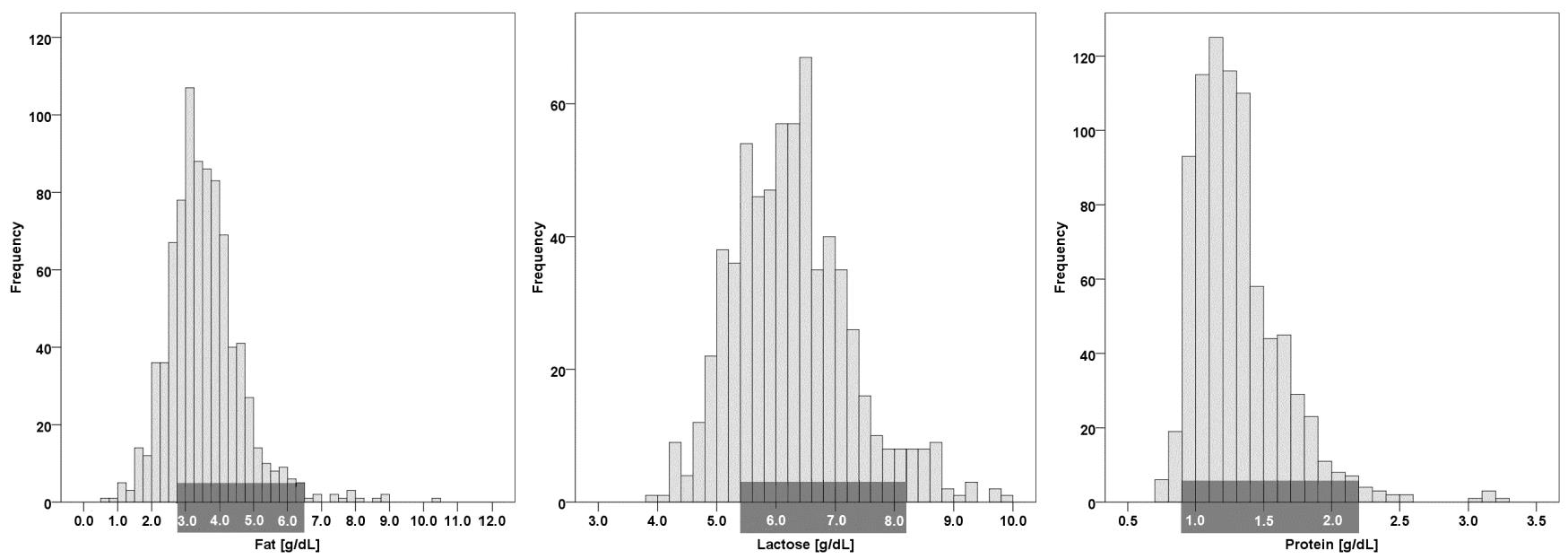


**Figure S2.** Macronutrient distribution (fat, lactose and protein) for all samples used in the calibration study (light grey) and coverage of the range by the samples of unpasteurized milk used for validation in this study (marked in dark gray).
